# The asthma mobile health study, smartphone data collected using ResearchKit

**DOI:** 10.1038/sdata.2018.96

**Published:** 2018-05-22

**Authors:** Yu-Feng Yvonne Chan, Brian M. Bot, Micol Zweig, Nicole Tignor, Weiping Ma, Christine Suver, Rafhael Cedeno, Erick R. Scott, Steven Gregory Hershman, Eric E. Schadt, Pei Wang

**Affiliations:** 1 Department of Genetics and Genomic Sciences, Icahn School of Medicine at Mount Sinai, New York, NY, USA; 2 Department of Emergency Medicine, Icahn School of Medicine at Mount Sinai, New York, NY, USA; 3 Center for Digital Health, Icahn Institute of Genomics and Multiscale Biology, Icahn School of Medicine at Mount Sinai, New York, NY, USA; 4 Department of Medicine, Stanford University, Stanford, California, USA; 5 Division of Cardiovascular Medicine, Department of Medicine, Stanford University, Stanford, California, USA; 6 Sema4, a Mount Sinai venture, Stamford, Connecticut, USA; 7 Sage Bionetworks, Seattle, WA, USA

**Keywords:** Clinical trial design, Research data, Asthma

## Abstract

Widespread adoption of smart mobile platforms coupled with a growing ecosystem of sensors including passive location tracking and the ability to leverage external data sources create an opportunity to generate an unprecedented depth of data on individuals. Mobile health technologies could be utilized for chronic disease management as well as research to advance our understanding of common diseases, such as asthma. We conducted a prospective observational asthma study to assess the feasibility of this type of approach, clinical characteristics of cohorts recruited via a mobile platform, the validity of data collected, user retention patterns, and user data sharing preferences. We describe data and descriptive statistics from the Asthma Mobile Health Study, whereby participants engaged with an iPhone application built using Apple's ResearchKit framework. Data from 6346 U.S. participants, who agreed to share their data broadly, have been made available for further research. These resources have the potential to enable the research community to work collaboratively towards improving our understanding of asthma as well as mobile health research best practices.

## Background and Summary

Smartphone usage has penetrated to over 80% of US households and with it comes a growing collection of wearables and embedded sensors. For example, passive location GPS tracking can be cross-referenced with external data sources such as Geographic Information System (GIS) data that can provide detailed and dynamic views of an individual’s environment^1^. Additionally, the maturity of online application discovery methods, such as Apple’s AppStore, makes it feasible to recruit patients into research studies remotely in a more cost-effective and efficient manner than traditional methods. Together, these factors have created an unprecedented opportunity to improve chronic disease management and simultaneously foster research to advance our understanding of wellness and disease.

Apple introduced ResearchKit in March 2015 as an open source software framework that enables a new mode of clinical research by leveraging the capabilities of the iPhone for remote, continuous, bi-directional transmission of data with individual research participants. The Asthma Health App (AHA) was one of the first of five ResearchKit apps that launched in March 2015 and was developed by the Icahn School of Medicine at Mount Sinai, in collaboration with Apple, Sage Bionetworks, and other partners^2^.

Asthma is one of the most common and costly chronic diseases, impacting 25 million Americans. In order to achieve optimal control of persistent asthma, patients should avoid exacerbating factors, monitor their symptoms, adhere to their prescribed treatment regimens, and adjust treatments during periods of worsening symptoms^3^. The AHA was designed to conduct large scale mobile health research by collecting continuous prospective, longitudinal data, while promoting self-education and monitoring, as well as providing real time feedback to the participants on their asthma status and protocol adherence^1^. Specifically, the AHA encouraged users to maintain an electronic asthma diary that tracked asthma symptoms and potential triggers. The AHA also provided automated medication reminders, and offered various optional educational videos on asthma as well as geographically specific weather and pollution information (using the iPhone GPS to determine coordinates of the user), allowing for correlation between factors identified in the environment and asthma symptoms. Moreover, based on user input, the app provided feedback regarding their asthma control using standard criteria (e.g. Global Initiative for Asthma score, etc.)^4^. The full working code for the Asthma Health app is open source and serves as a template for others in designing mobile health apps using ResearchKit.

Using Asthma Health App, we successfully conducted the asthma mobile health study between 3/9/2015 and 11/30/2016. Participants were continuously enrolled throughout the study period. Encouraging results based on data from the first six months of this study were reported in Chan *et al*.^2^. In this paper, we describe and make available the data from the complete (~21 months) study period. We are hopeful that the research community will leverage the resources provided here to further the collective mission of improving asthma care and advancing digital health research.

## Methods

### Participant onboarding

After the AHA was released in the Apple App Store on March 9th 2015, the application was downloaded 58,182 times over the 21 month study period, 48 054 of which were from participants in the U.S. Prospective participants could download the app, walk themselves through the eligibility screening (18 years of age or older, have asthma, not pregnant and literate in English), and then proceed through the rest of the e-consent process.

**Figure 1:**
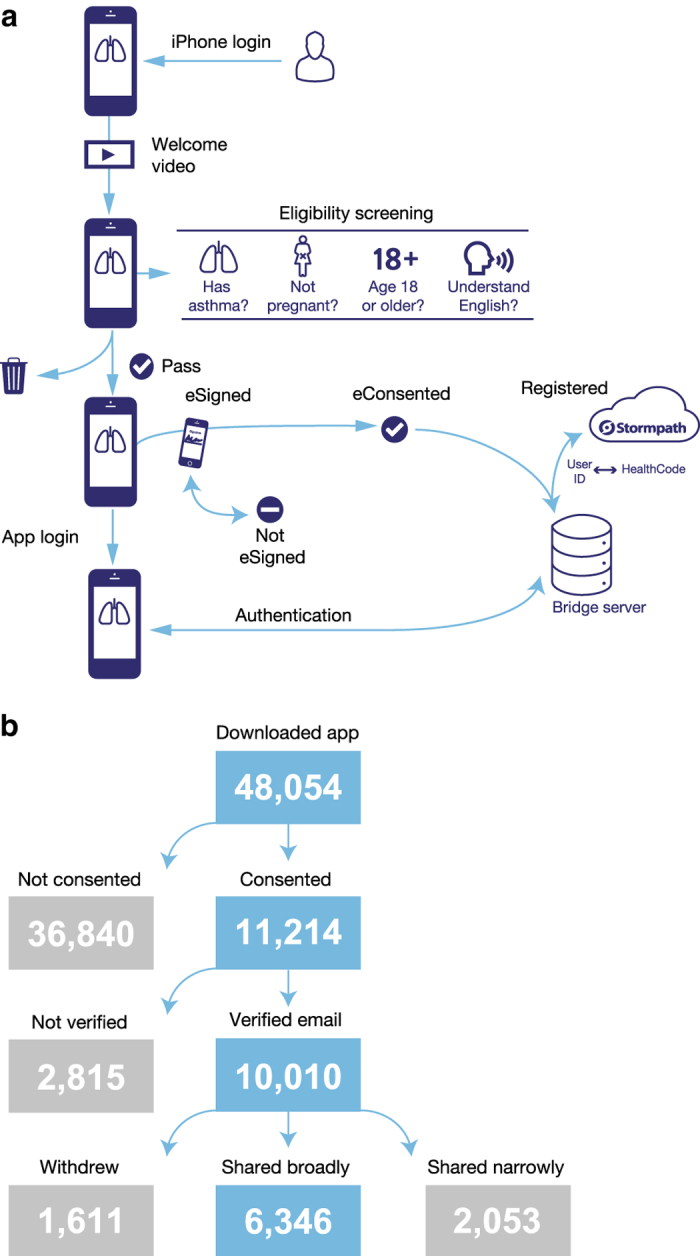
User experience and study sample sizes. (**a**)Diagram of user experience. (**b**) Flowchart describing sample sizes during onboarding process.

Ethical oversight of the study was obtained from the Icahn School of Medicine at Mount Sinai IRB (GCO#15-0063). First, the user learned about the study details from a series of mandatory consent screens. Prior to electronically signing the informed consent form, the user had to have passed a comprehension quiz to ensure they understood key elements of the consent screens including risks and benefits of the study, data sharing options, and privacy protection. Participants were obligated to make an active choice to complete the consent process (no default choice was presented). After they signed the consent form, they provided an email address to both authenticate their identity and to receive a PDF copy of their signed consent form. The user was required to verify their email address by clicking on a link within an email sent by the Sage Bionetwork’s servers. This served to ensure a method of contact with the user and recovery of account information should the user switch to a different iPhone. Figure 1a provides a simplified layout of the initialization process in the Asthma Mobile Health Application study.

Moreover, participants were given the option to share their data either solely with the Mount Sinai study team and partners (‘share narrowly’) or more broadly with qualified researchers worldwide (please see Supplementary Figure 1 for the screenshot of this option). Participants were also provided with an option of "leave study" in the AHA to allow them withdraw from the study anytime during the study period.

The data presented here were contributed by individuals who were US resident and chose to share their data broadly. Figure 1b describes the sample size of users during onboarding process. A total of 10 010 users consented to participate in the study and provided a verified email address for enrollment, of which 1611 participants later withdrew from the study. Of the remaining 8399 participants, 6346 (76%) opted to sharing their data broadly with qualified researchers worldwide. Of these, 5875 submitted task or survey data. It is this cohort that is herein described in this paper.

### Study survey design

We were unable to directly use certain standard validated asthma surveys in our study due to licensing constraints^2^. Instead, our asthma specialists in the Mount Sinai health system developed AHA surveys by incorporating general content used by validated survey instruments. Specifically, medical history survey were created based on Charlson Comorbidities Index; daily and weekly surveys were created to be able to capture symptoms to assess disease severity and control; and EQ-5D-5L is validated general quality of life survey (though this is first time it’s on a modern smartphone).

### Active data collection (surveys)

Much of the AHA data was collected via a series of surveys that were scheduled to appear on the participant’s ‘Activities’ screen. Baseline surveys appeared successively over the first 3 days after enrollment. These surveys were designed to collect data on: 1) asthma history, including the frequency and time (day or night) of symptoms and the extent of activity limitation due to symptoms; 2) asthma experience, including triggers and personal management plans, 3) medical history (prior and current disease and allergies); and 4) demographics (ethnicity, race, age, gender, income, and education) [Data Citations 1, 2, 3, 4, 5, 6]. Additionally, baseline information, such as healthcare utilization, asthma medications, self-reported controller adherence, quick relief/rescue medication use, peak flow, and other clinically relevant data were collected. Participants were also asked to identify what troubles them about their asthma, and were prompted to set a goal for asthma control. Lastly, participants were asked to complete the EQ-5D-5 L (EuroQol version 5D-5 L), which assesses health related quality of life [Data Citation 7]. After the aforementioned 3-day initialization process, users were asked to complete additional daily and weekly surveys [Data Citations 8, 9], with EuroQol, 6-month milestone and app feedback surveys [Data Citation 10], occurring at less frequent intervals. Please see Supplementary Table 1 for detailed survey questions. The data obtained from these surveys is subject to the copyright holder’s license.

### Passive data collection

The AHA passively collected locations using the device’s GPS and linked it, along with air-quality reports, to the study data. In the initial release of the app, the participant’s nearest EPA air-quality reading along with that station’s city and state were sent when the participant viewed the dashboard tab. However, since the location information of weather stations does not provide satisfactory accuracy or resolution, as of version 1.0.6 released on May 5 2015, the app started to hourly collect raw latitude and longitude based on the device’s GPS whenever the user’s location changed. In this data release, the daily 3-zip-code location information of each user after May 5 are provided [Data Citation 11].

### Data storage and security

The app recorded all data collected for this study through interactions with Bridge Server, a set of web services developed and operated by Sage Bionetworks. Supplementary Fig. 7b in Chan *et al.*^2^ provides a detailed description of the backend design on health data encryption.

Bridge is a technology platform designed to support biomedical research studies conducted through smartphones and other sensor devices. Bridge services support mobile registration and consent to participate in research studies, the design and scheduling of surveys and mobile sensor-driven activities, and the receipt of sensor and survey data from mobile sources. These services have been used broadly to support a variety of health studies, including all five of the initial Research Kit apps launched in March 2015 (refs 5–7).

Coded study data, consisting of survey responses and GPS coordinates, were exported to Synapse for distribution to researchers. Synapse, a general-purpose data and analysis sharing service, enables researchers to work collaboratively to analyze data and share insights. Synapse was developed and operated by Sage Bionetworks as a service to the biomedical research community^5^.

Because of an initial technical issue with the integration of HealthKit and ResearchKit data, demographic information is missing from a number of participants. Multiple versions of the AHA were released during the study period to address these software-related concerns and to implement new features (see Supplementary Table 6 in Chan *et al.*^2^).

### Code availability

The Asthma Health App version 1.011 (https://github.com/ResearchKit/AsthmaHealth) was built using Apple’s ResearchKit framework (http://researchkit.org/), which is open source and available on GitHub (https://github.com/researchkit/researchkit). AppCore (https://github.com/ResearchKit/AppCore) is a layer built on top of ResearchKit that was shared among the five initial ResearchKit apps. The Bridge iOS SDK (https://github.com/Sage-Bionetworks/Bridge-iOS-SDK) provides integration with Sage Bionetworks’ Bridge Server, a back-end data service designed for collection of participant donated study data (https://developer.sagebridge.org/).

### Limitations

Given the Asthma Mobile Health study (AMHA) is conducted via iPhones, the study population reflects its users and introduces socioeconomic bias. A 2016 Pew research study found that iPhone owners have higher education levels and income than other smartphone users, who as a group have higher income and education levels than the general population. “Only 5% of AHA users with asthma are Black, compared to 13% of the US population, an underrepresentation commonly encountered in clinical research in general. In the United States, 92% of Hispanics, 91% of Whites, and 94% of Blacks report using a mobile phone, with 64% of Hispanics, 66% of Whites and 64% of Blacks using a smartphone”^2,8^. Therefore, the use of study app on the Android platform may capture more diverse groups. Additionally, the combination of traditional and digital research methods may be maximally effective in enrolling participants that most closely represents the general population^2^.

One novel aspect of this study dataset is its link between Asthma symptoms and geography. For the subset of participants who consented to share their geolocation data with qualified researchers, we report their time course geographic data of 3 digit zip codes. Note, during the electronic informed consent process, participants agreed to share data with the understanding that their personally identifiable information (PII)/ protected health information (PHI) removed. Thus, more refined geographic information, such as full zip code or longitudes/latitudes are not released.

The symptom data generated in this smartphone study are based on self-reported surveys. Lee *et al* (2003) noted that different methods of defining asthma severity provide different distribution of patients across categories of disease (mild to severe). However, our study methodology did not differ from common practice in most asthma epidemiologic studies, where symptom-based surveys were used without corresponding biometric measurements (i.e. lung function)^2^.

Lastly, we observed significant decline in study retention over time. As discussed in Chan *et al.*^2^, the observed dropoff in user retention is shared by multiple ‘digital’ use cases (e.g., mobile apps including entertainment ‘gaming’ apps, tutorial videos, open online courses) and thus suggests the biopsychosocial tendencies and behaviors of users may be hardwired. Given the ultimate goals of digital health generally rely on prolonged participation, the creators of these tools must devote attention and resources to incorporating psycho-social and behavioral principles in digital health design beyond technical ones. One consideration is to offer financial incentives for study participation to enhance retention- which is standard practice in clinical research^9,10^.

Given the aforementioned limitations and learnings from the AMHS, we believe research hypotheses with the following characteristics are appropriate for the current ResearchKit methodology: minimal risk clinical studies, allowing the use of electronic consent, requirement for rapid enrollment across diverse geographical locations and frequent data collection, a hypothesis that can be answered within a short time period, data collection that is passive (GPS, physical activity, etc.), no assumption that results will be generalizable to participants recruited via traditional methods, and a sample size and statistical analysis plan that account for the known attrition/missing data historically seen in internet/mobile app studies^2^.

## Data Records

All coded data sets are stored and accessible via the Synapse platform in a public project with associated metadata and documentation (https://www.synapse.org/asthmahealth). Please see Table 1 for a summary on survey contents and releasing frequencies, as well as numbers of surveys and activities completed by study participants who agreed to share their data broadly [Data Citations 1, 2, 3, 4, 5, 6, 7, 8, 9, 10, 11].

Table 2 illustrate the demographic and baseline clinical characteristics of study participants [Data Citations 1, 2, 3, 4, 5, 6] and compared with population-based asthma statistics from the 2015 Behavioral Risk Factor Surveillance System (BRFSS) of the Centers for Disease Control and Prevention (http://www.cdc.gov/asthma/most_recent_data.htm). AHA users tended to be younger, wealthier, more educated, and were more often male than asthma patients in the CDC asthma population. Note, control of asthma symptoms was assessed according to Global Initiative for Asthma (GINA) based on the number of the following statements about a patient’s asthma symptoms that are true: 1) daytime symptoms occur less than twice per week (<8 times per month); 2) no occurrence of nocturnal awakenings (0 per month); 3) quick relief of symptoms occurs fewer than twice per week (<8 puffs per month); and 4) no activity limitation due to asthma symptoms (0 per month). Asthma is considered ‘Uncontrolled’ if four of the above statements is true, ‘Partly controlled’ if 2–3, and ‘Well controlled’ if 0–1 (http://ginasthma.org/).

**Figure 2:**
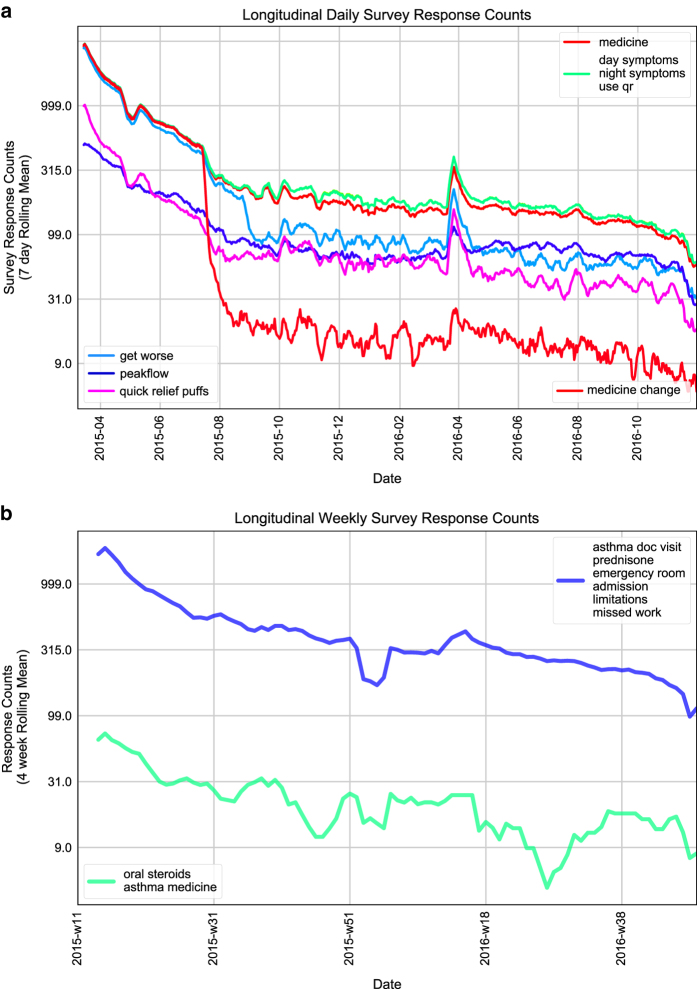
Longitudinal response counts to daily and weekly survey questions. (**a**) 7 day rolling mean of daily survey question response counts across participants. (**b**) 4 week rolling mean of weekly survey question response counts across participants. The survey question labels used in the figure legends correspond to the column names of survey data matrices, and are annotated in Supplementary Table 1. Substantially overlapping curves were plotted with a single line and grouped in the legend. Y-axis is plotted on a log10 scale with y-axis ticks displaying back-transformed counts.

**Figure 3:**
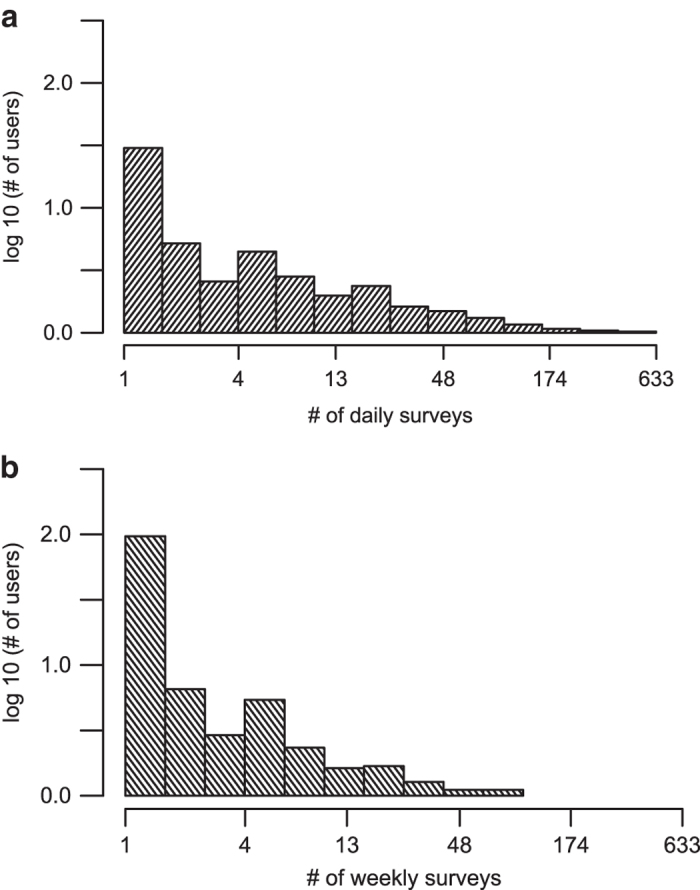
Individual response counts to daily and weekly surveys. (**a**) Individual response counts of daily surveys. (**b**) Individual response counts of weekly surveys.

**Figure 4:**
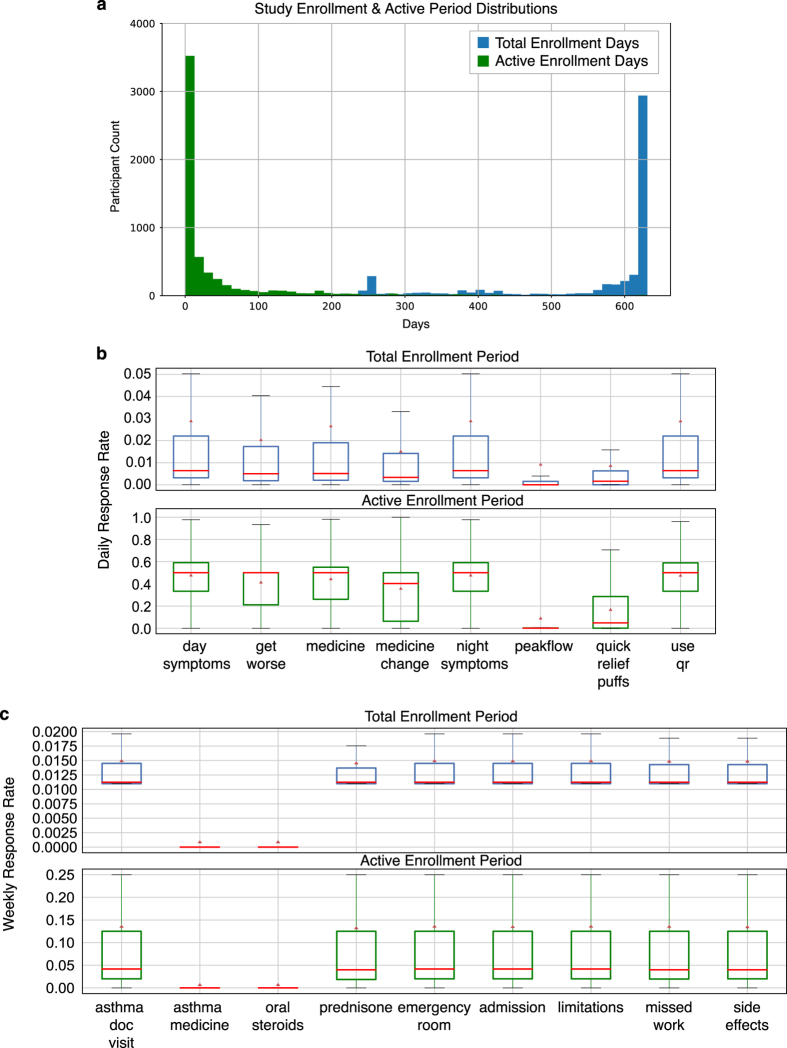
Distributions of enrollment lengths and individual response rates. (**a**) Distributions of participants’ total enrollment lengths (blue) and active enrollment lengths (green). (**b**) Boxplots of individual response rates of daily survey questions, which are ratios of numbers of days with non-null responses of that question to either total enrollment lengths (top) or active enrollment length (bottom) of participants. (**c**) Boxplots of individual response rates of weekly survey questions, which are ratios of numbers of weeks with non-null responses to either number of weeks during total enrollment period (top) or active enrollment period (bottom) of participants. The survey question labels used in the figure legends correspond to the column names of survey data matrices, and are annotated in Supplementary Table 1.

The numbers of responses to each question in the daily and weekly survey on each study day [Data Citations 8, 9] are illustrated in Fig. 2. The distributions of the total number of daily or weekly surveys provided by one user during the study period [Data Citation 8, Data Citation 9] are illustrated in Fig. 3. Distribution of total enrollment lengths as well as active enrollment lengths of study participants are shown in Fig. 4a. Note, the total enrollment length of one participant was defined as the number of days between the timestamp of his/her first survey and December 1, 2016; while the active enrollment length was defined as the number of days between the timestamps of one’s first and last survey responses. In addition, Fig.s 4b and c illustrate the boxplots of individual response rates to each question in the daily and weekly surveys. Specifically, individual response rate of one participant to a daily (or weekly) survey question is the ratio of the number of days with at least 1 non-null response to this survey question within a 1 day (or 7 day calendar week) period to the numbers of days or weeks during either the total or active enrollment periods. We also want to mention that there were 1047 and 22 instances of a participant submitting 2 and 3 weekly surveys within 1 calendar week, respectively [Data Citation 9].

**Figure 5:**
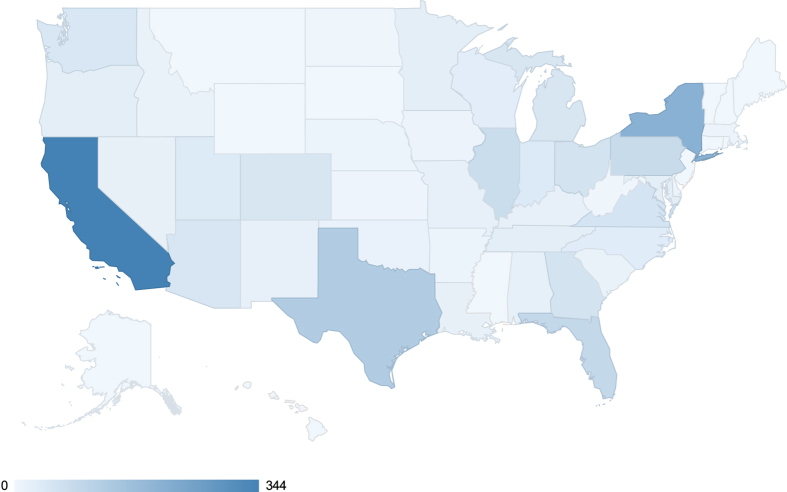
Geographic distribution. The map illustrates the geographic of 1959 participants who agreed to share their data broadly and supplied location data after May 5, 2015.

For the 1959 participants who supplied location data (after May 5, 2015), we illustrate their geographic distribution [Data Citation 11] by state in Fig. 5. The three states with the largest number of participants are California, New York and Texas.

## Technical Validation

The data provided herein are participant reported outcomes in the form of survey responses and should be treated as such.

In consultation with data governance experts, outlier values for height (<60 or >78 inches) and weight (<80 or >350 pounds) were censored to protect individuals who may have uniquely identifiable traits. In addition, for one-time surveys, occasionally multiple versions were completed due to re-installation of the app. For these cases, the first survey that was filled out was reported.

In addition, in our app, we purposely eliminated default selections in all survey questions to avoid any biases caused by this mechanism. Other features that help to reduce fraudulent response include allowing users to skip any survey questions, and only showing one question per page (straightlining is common when answers are presented in matrix form).

We also assess the quality and validity of the study data obtained from this mobile health (ResearchKit) research method, which was discussed in detail in our Nature Biotechnology publication (excerpt below)^2^. Validity of the study data was supported by concordance between our cohort’s self-reported asthma status at baseline and prospectively collected data. For example, participants’ daily survey responses for day and night symptoms as well as inhaler and controller medication usage were all found to be significantly associated with the GINA control levels calculated based on intake questionnaires for these parameters. Peak flow measurements submitted by participants were of expected range based on known trends for patients’ sex, height, and asthma control status. We detected that patients’ asthma symptoms correlated well with the frequency of rescue inhaler usage and peak flow values as would be expected based on the clinical behavior of asthma. Likewise, the self-reported asthma triggers (e.g., pollen, extreme temperature, air quality, pollutant exposures) mapped based on geography and time correlated well with objective measures (e.g., external, validated environmental sources).”^2^.

## Usage Notes

Due to the novel nature and collection method for these data, governance structures have been put in place in order to respect the balance between the desire of participants to share their data with qualified researchers and the respect for privacy of those participants. Researchers who are interested in accessing these data need to complete the following steps:

1. Become a Synapse Certified User with a validated user profile
2. Submit a 1–3 paragraph Intended Data Use statement. Note that your Intended Data Use statement will be posted publicly on Synapse
3. Agree to comply with the data-specific Conditions for Use when prompted:
4. ○ You confirm that you will not attempt to re-identify research participants for any reason, including for re-identification theory research.
5. ○ You reaffirm your commitment to the Synapse Awareness and Ethics Pledge.
6. ○ You agree to abide by the guiding principles for responsible research use and data handling as described in the Synapse Governance documents (http://docs.synapse.org/articles/governance.html).
7. ○ You commit to keeping these data confidential and secure.
8. ○ You agree to use these data exclusively as described in your submitted Intended Data Use statement.
9. ○ You understand that these data may not be used for commercial advertisement or to re-contact research participants
10. ○ You agree to report any misuse or data release, intentional or inadvertent to the ACT within 5 business days by emailing act@sagebase.org
11. ○ You agree to publish findings in open access publications.
12. ○ You promise to acknowledge the research participants as data contributors and the study investigators on all publication or presentation resulting from using these data as follows: *"These data were contributed by users of the asthma app as part of the Asthma Mobile Health Application study developed by The Icahn School of Medicine at Mount Sinai and described in Synapse [doi:10.7303/syn8361748]."*

See the full instructions for requesting data access on the Accessing the Asthma Data page (https://www.synapse.org/#!Synapse:syn8361748/wiki/415364).

## Additional information

**How to cite this article:** Chan, Y.-F.Y. *et al.* The asthma mobile health study, smartphone data collected using ResearchKit. *Sci. Data* 5:180096 doi: 10.1038/sdata.2018.96 (2018).

**Publisher’s note:** Springer Nature remains neutral with regard to jurisdictional claims in published maps and institutional affiliations.

## Supplementary Material



Supplementary Figure 1

Supplementary Table 1

## Figures and Tables

**Table 1:** Summary for each survey and activities completed by study participants.

Task Name	When & Frequency?	Content	Data Citation	Unique Participants	Unique Records
Demographics Survey	Appears on first day	Demographics are collected (height, weight, gender, age)	Data Citation 1	2593	2593
Your Asthma Survey	Appears on second day	Questions about triggers, flu shot, asthma action plan, spirometer, asthma troubles and goals	Data Citation 2	3849	3849
Medical History Survey	Appears on fourth day	Questions about comorbidities (heart disease, lung disease, allergies, cancer, etc.)	Data Citation 3	3251	3251
Asthma Medication Survey	Appears on first day, Reappears on day 165 (updated survey)	All asthma medications (Controller & Rescue) with their doses	Data Citation 4	5174	5174
Asthma History Survey	Appears on first day	Variety of questions about asthma history including those required to compute baseline GINA	Data Citation 5	5476	5476
About You Survey	Appears on third day	Demographics (ethnicity, race, income, education), smoking status, health insurance.	Data Citation 6	3728	3728
Daily Prompt Survey	Recurs daily	Symptoms, rescue inhaler usage, controller inhaler compliance, triggers, peak flow	Data Citation 7	5286	75,795
Weekly Prompt Survey	Recurs every 7 days	Questions about health care utilization, activity limitations, missed work and side effects	Data Citation 8	2449	13,614
EQ5D Survey	Appears on first day, Recurs every 90 days	Questions about mobility, self-care, usual activities, pain/discomfort, anxiety /depression and overall score (0-100)	Data Citation 9	3445	4617
Milestone Survey	Appears on day 165	Demographics are recollected (height, weight, gender, age), repeat of questions from Asthma History including questions to compute GINA, ratings, feedback, (least) favorite feature, lessons learned, etc.	Data Citation 10	212	212
Participant 3 Digit Zip	Every time the app’s dashboard (task/survey page) is accessed	Based on GPS location (latitude, longitude)^a^	Data Citation 11	1959	24,190

^a^To remain within the regulations of our IRB protocol regarding PII, we report the 3 digit zip code instead of GPS coordinates.

**Table 2:** Clinical and demographic characteristics of AHA users.

Characteristic		Count	AHA %Dist.	CDC %Dist.
Age^a^	18-34	1553	60%	30%
	35-64	930	36%	54%
	65+	85	3%	17%
	NA	3307	NA	—
Gender^a^	Female	1001	39%	66%
	Male	1564	61%	34%
	NA	3310	NA	—
Race	Black	163	5%	14%
	White	2419	69%	67%
	Other	247	7%	6%
	Multi	165	5%	—
	Hispanic	501	14%	12%
	NA	2380	NA	—
Smoking Status	Never	2885	78%	—
	Current	180	5%	21%^c^
	Former	638	17%	—
	NA	2172	NA	—
Age of diagnosis	<=18 years of age	4277	80%	—
	>18 years of age	1084	20%	—
	NA	514	NA	—
Asthma Control Medication	Yes	3461	67%	—
	No	1547	30%	—
	Not Sure	146	3%	—
	NA	721	NA	—
Daily Inhaled Medicine	ICS/LABA	2003	65%	—
	ICS	1093	35%	—
	NA	2779	NA	—
GINA	Uncontrolled	2349	46%	50%^d^
	Partly Controlled	1937	38%	—
	Well Controlled	821	16%	—
	NA	768	NA	—
CDC Demographic data was obtained from the 2015 Behavioral Risk Factor Surveillance System (BRFSS) (https://www.cdc.gov/asthma/most_recent_data.htm). Based on 5875 AHA users who submitted survey data.				
^b^ Defined as having smoked less than 100 cigarettes in lifetime				

^a^Data from the 6-month milestone survey was used for users who did not report their age gender at baseline.

^c^Instead of using the GINA criteria, the CDC used a slightly different criteria to define uncontrolled asthma patients as those who reported any of the following: (1) asthma symptoms more than two days a week in the past 30 days, (2) nighttime awakenings for more than one time a week in the past 30 days, or (3) short-acting β2-agonists use more than two days a week in the past three months. Source: http://www.cdc.gov/asthma/asthma_stats/uncontrolled_asthma.htm

^d^Source: https://www.cdc.gov/asthma/asthma_stats/people_who_smoke.htm.
