# Supplementary figures and images for: The asthma mobile health study, smartphone data collected using ResearchKit
Source: Sci Data. 2018 May 22;5:180096. doi: 10.1038/sdata.2018.96 (PMC5963336; doi:10.1038/sdata.2018.96)

**Supplementary Figure 1.** Screenshot of sharing options page.


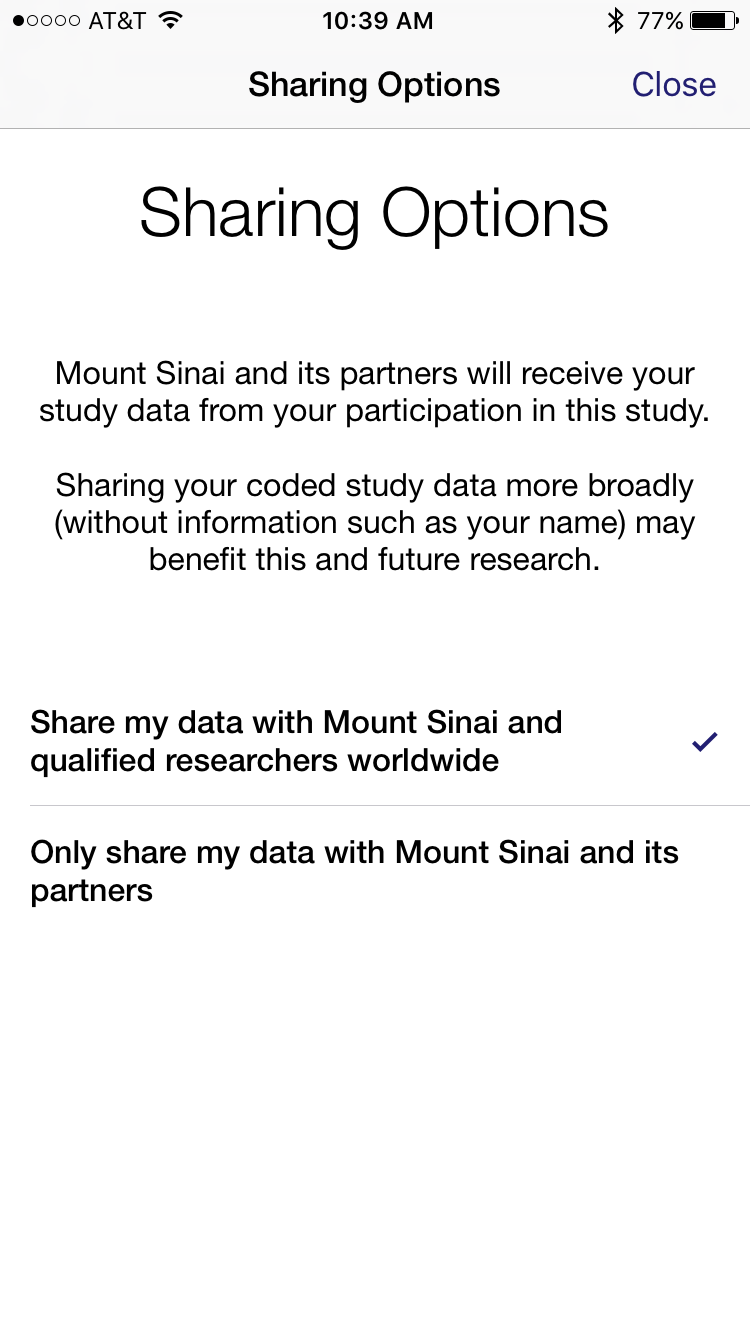

Supplement: Supplementary Figure 1 [file sdata201896-s2.docx]
